# Beyond average: potential for measurement of short telomeres

**DOI:** 10.18632/aging.100462

**Published:** 2012-06-08

**Authors:** Elsa Vera, Maria A. Blasco

**Affiliations:** Telomeres and Telomerase Group, Molecular Oncology Program, Spanish National Cancer Centre (CNIO), Madrid, E-28029, Spain

**Keywords:** telomere length, short telomeres, quantitative FISH, telomeric southern blot, quantitative PCR

## Abstract

The length of telomeres, and in particular the abundance of short telomeres, has been proposed as a biomarker of aging and of general health status. A wide variety of studies show the association of short telomeres with age related pathologies and cancer, as well as with lifespan and mortality. These facts highlight the importance of measuring telomere length in human populations and by using reliable methods to uncover the association between telomere length and human disease. This review discusses the advantages and drawbacks of current telomere length measurement methods. Most of these methods provide mean telomere length values per cell or per sample and very few of them are able to measure the abundance of short telomeres, which are the ones indicative of telomere dysfunction. The information provided by each method and their suitability for different studies is discussed here.

## INTRODUCTION

Telomeres are special structures at the ends of eukaryotic chromosomes that protect them from degradation and DNA repair activities [1]. In vertebrates, telomeres are composed of tandem repeats of the TTAGGG sequence bound by a set of specialized proteins[1]. In the absence of a compensatory elongating mechanism, telomeres get shorter with each cell division owe to the so-called end replication problem [2]. Telomerase is a reverse transcriptase that can elongate telomeres *de novo* during cell division [3, 4]. After birth, telomerase is silenced in most of somatic cells and telomeres progressively shorten with aging [2, 5]. Certain cell types, such as cells of the hematopoietic lineage, stem cells, and germ cells, have the ability to activate telomerase, but this is not sufficient to prevent telomere shortening with aging [6, 7]. Critically short telomeres cannot be repaired by any of the known DNA repair mechanisms and consequently trigger a persistent DNA damage response (DDR), which leads to cellular senescence and/or apoptosis [8, 9], eventually compromising tissue regenerative capacity and function, and contributing to organismal aging (Figure 2)[10]. Genetic studies in mice have demonstrated that short telomeres rather than average telomere length (TL) are causative of age-related pathologies and that recue of short telomeres by telomerase is sufficient to restore cell and organismal viability as well as genomic stability [11, 12]. For this reason, the frequency of short telomeres, rather than the mean telomere length (TL), is proposed to be a better indicator of telomere dysfunction and, consequently, of cell and tissue dysfunction. This highlights the relevance of measuring the frequency of short telomeres in addition to mean telomere length in human population studies. However, the vast majority of human population studies performed to date only rely on the mean telomere length of a given tissue sample, usually blood cells. These studies have uncovered an association between lower values of mean telomere length and an increase risk to develop several-age related diseases, such as cardiovascular pathologies [13-15] (Figure 2) and even with lifespan and mortality [16-18]. In this regard, a recent publication described that telomere length early in life is a strong predictor of lifespan in zebra finches [18]. Having shorter telomeres than normal has been also related to an increased risk of different cancers [19, 20]. More recently, the measurement of the percentage of cells with short telomeres or the abundance of short telomeres per nuclei has also been introduced to human population studies revealing new associations between higher frequency of short telomeres and cognitive impairment [5] or even with mood disorders such as bipolar disorder type II [21] (Figure 2). Thus, telomere length, and in particular, the abundance of critically short telomeres is likely to become a useful biomarker of aging and age-related diseases.

**Figure 1:**
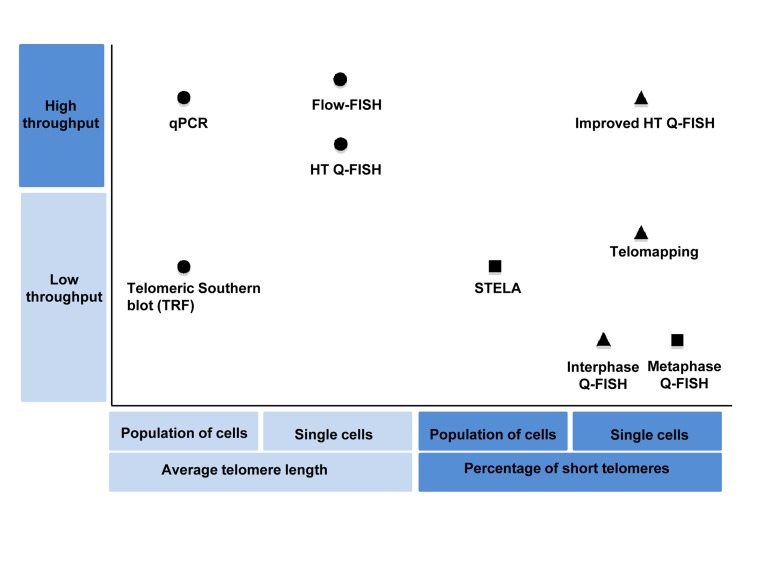
Classification of the main telomere length measurement methods according their throughput and the telomere length associated variable measured by them Shapes indicate the type of telomere length associated variable measured by each method: Circles represent method that measure average telomere length, triangles represents methods that are able to measure individual telomere spots and squares represent those that are able to measure individual telomeres.

**Figure 2:**
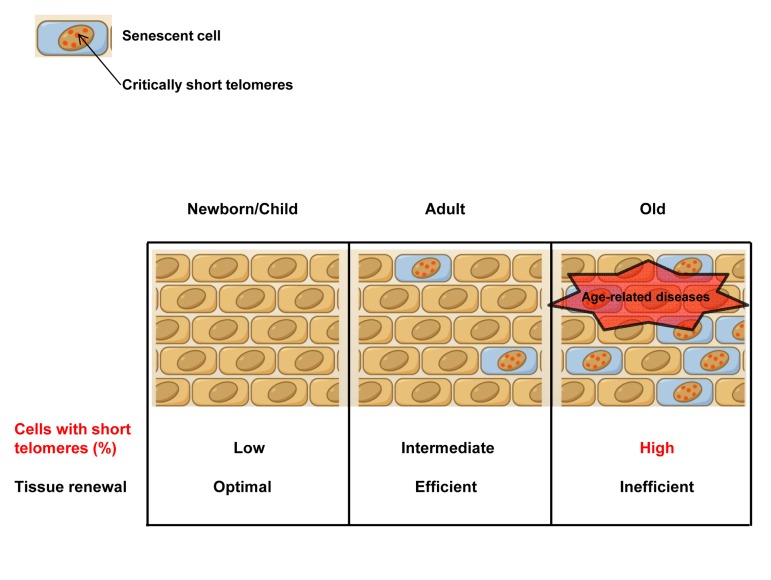
Accumulation of cells with short telomeres with aging In old individuals, the accumulation of apoptotic/sencescent cells with critically short telomeres as well as the loss of the regenerative potential of the stem cell pools as a consequence of telomere shortening, compromise tissue function and tissue regenerative capacity, contributing to organism aging. For this reason, the frequency of short telomeres, rather than the mean TL, is determinant for telomere dysfunction, and, consequently, for cell and tissue dysfunction. This fact points out the importance of measuring the frequency of short telomeres rather than the mean telomere length.

Telomere length studies were started around 1980s and were initially based on Southern blot quantification of a smear of telomere restriction fragments, the so-called terminal restriction fragment (TRF) analysis [22-24]. TRF analysis provides a rough estimate of the average number of telomeric repeats per sample, as terminal restriction fragments include not only telomeric repeats but also variable amounts of subtelomeric sequences. In spite of its limitations, the use of TRF analysis was instrumental to unveil the firsts associations of telomere length with aging and human diseases and still remains as one of the most widely used methods for telomere length analysis. In the 1990s, the development of telomere measurement methods based on telomeric Fluorescence In Situ Hybridization (FISH) solved the problem of accuracy and represented a breakthrough for telomere length quantification [25]. The first telomere FISH based method developed was the so-called telomere quantitative FISH method or Q-FISH on metaphases (also known as conventional Q-FISH), which allows telomere length quantification of individual chromosome ends at the single-cell level, providing a quantification of both very short/undetectable telomeres and of the mean telomere length per cell [25]. To date, Q-FISH on metaphases remains as one of the most accurate and sensible methods available to measure individual telomeres per nuclei in a cell population. However, the conventional Q-FISH method is very time consuming and labor intensive, and therefore, not suitable for high-throughput (HT) assays. In this regard, a medium-throughput telomere Q-FISH method based on flow cytometry was later developed, known as Flow-FISH, however, this methodology only provides values of mean telomere length per cell, and therefore is less accurate than conventional Q-FISH [26, 27]. More recently, we have developed an automated HT quantitative telomere FISH platform, which we named HT quantitative FISH (HT Q-FISH), that allows highthroughput quantification of both mean telomere length and the percentage of short telomeres per cell in large human population studies [5]. In addition, to the FISH-based telomere quantification methods, the telomere quantitative PCR (qPCR) is quite widely used as it does not require live cells and its fast and highthroughput [28]. However, as important drawbacks are the fact that only renders mean telomere length values per sample and does not allow the quantification of short telomeres. These limitations have been circumvented by a PCR-based method known as single telomere analysis (STELA), which allows quantification of specific chromosome ends, although STELA is not amenable for highthroughput studies. A detailed comparison between these different methodologies and the information they provide is discussed in this review.

### Telomere Restriction Fragment (TRF) Analysis

As mentioned above, TRF remains as the most widely used technology to measure telomere length [22-24] (Table 1). TRF relies on the differential digestion of genomic DNA using frequently cutting enzymes, which will release a termimal restriction fragment cointaining the telomeric repeats, which do not contain sites for restriction enzymes, and variable lengths of subtelomeric sequences. These big DNA fragments are separated by gel electrophoresis, and hybridized with a radioactive telomeric probe by Southern blot. A smear of terminal restriction fragments (TRFs) is typically obtained which contains telomeric DNA of variable lengths as well as 2.5-4 kb of subtelomeric DNA [29, 30]. The average size of TRFs, as indicated by the migration distance of gel bands, provides an estimation of the mean telomere length of the sample (Table 2 and Figure 1).

**Table 1:** Summary of the advantages and drawbacks of the main telomere length measurement methods

Method	Approach	Advantages	Drawbacks
**Telomere Restriction Fragment (TRF)**	Southern blot hybridization using probes against telomere repeats.	-Well known and widely used technique. -It has no special requirements	-Difficult to quantify. -Requires many cells (~105). -Provides and estimate of the average telomere length per sample -Subtelomeric polymorphism.
**Telomere measurement by quantitative PCR**	It measures the ratio of telomere repeat copy number to single copy gene copy number.	-Simple -Fast -Scalable to achieve a high throughput (HT) of samples.	-It quantifies the average telomere length per sample and cannot quantify individual telomeres.
**Flow FISH**	Based on the determination of telomere fluorescence in individual interphase cells using fluorescence-activated cell sorting (FACS) technology.	-Simple. -Amenable to automatization, -Quantitative, reproducible, and accurate.	-Restricted to isolated cells, and cell suspensions. -Requires expensive and technical demanding system. -Not many samples (<20) are processed and analyzed at the same time. -It quantifies the average telomere length per cell.
**Metaphases quantitative FISH**	Based on the use of digital fluorescence microscopy to determine telomere fluorescence after hybridization of metaphase spreads with a fluorescent PNA telomeric probe.	Permits the measurement of telomere length at each individual chromosome end. -Allows quantification of the number of “signal-free ends” (<0.15 kb) -High accuracy.	-Labor-intensive and time consuming (week/s) -Requires expensive and technical demanding system. -External calibration (from auf to kb) -Many controls required to avoid inter/intra-session variability. -Very few samples are analyzed at the same time.
**Single Telomere Length Analysis (STELA)**	It is a ligation PCR-based method.	-It requires no specialized equipment. - It requires very limited starting material	-It is usually restricted to several well characterized chromosome ends: XpYp, 2p, 11q and 17p. -It is limited in the analysis of long telomeres (typically >20kb). -Labor intensive and low throughput.

**Table 2:** Main telomere length measurement method characteristics

TL associated variables	Method	Short telomeres stimation	Resolution	Cell number required	Throughput	References
**Mean TL per sample**	**TRF** **qPCR**	No No	1kb ND	1×10^6^ cells 20 ng DNA	Low High	22-24 28
**Mean TL per cell**	**Flow-FISH**	No	0.3kb	0.5×10^6^ cells	High	26
**Individual telomere spots**	**Interphase Q-FISH** **HT Q-FISH (Improved method)** **Telomapping**	Yes Yes Yes	0.3kb 0.3kb 0.3kb	30 interphases 0.01×10^6^ adherent cells 0.07×10^6^ lymhoid cells Fixed hystologic section	Low High Low	37 5 (Original) 21, 40 (Improved) 6
**Individual telomeres**	**Q-FISH** **STELA**	Yes Yes	0.3kb 0.3kb	15-20 metaphases 0.1×10^6^ cells	Low Low	25, 33 45

ND, not determined

The TRF analysis has numerous drawbacks (Table 1). First, the method does not yield information on individual chromosome ends but only an estimation of average telomere length per sample. Second, the methodology is time-consuming and requires large number of cells (~106). Third, the size of TRFs are only an estimate of the length of telomere repeats as they contain subtelomeric repeats, which can vary in length depending on the last restriction site at a given chromosome arm, thus increasing the heterogeneity of the TRFs and masking the real length of telomeric repeats. Fourth, Southern analysis underestimates the length and number of short telomeres in the sample since short fragments disperse over a large distance in the gel compared to the longer fragments and furthermore provide lower hybridization signal. Fifth, it requires pulse field gel electrophoresis to elucidate long telomere size such as mice telomeres. Seventh, the TRF methodology requires handling of radioactivity. Lastly, quantification of smear patterns in autoradiograms is a quite error-prone method. In summary, under-estimation of short telomeres together with the fact that TRF cannot measure telomeres in individual cells, are important drawbacks for the use of TRF in aging and longevity studies.

### Fluorescence *In Situ* Hybridization (FISH) Methods

The development of FISH methods to measure telomere length successfully solved some of the drawbacks of the TRF method, providing increased sensitivity, specificity, and resolution [25] (Table 1). The main achievement of the FISH-based methods relies on their ability to measure telomere length at the single-cell level (Q-FISH on interphasic cells, Flow-FISH, and HT Q-FISH) or even at the level of individual telomeres per chromosome (Q-FISH on metaphases), both using cell suspensions or histological sections (Table 2, Figure 1).

Telomere length analysis by FISH is based on the specific labeling of telomeres with fluorescent peptide nucleic acid (PNA) oligonucleotide probes [25]. PNA probes are synthetic peptides homologous to DNA, in which the negatively-charged phosphate-pentose backbone of the DNA is replaced by an uncharged N-2 amine ethyl-glycine backbone [31]. This modification yields an extraordinarily stable, efficient, and specific hybridization of the probe to the target DNA [32]. Each PNA probe recognizes three telomeric repeats (T2AG3). For this reason, the intensity of the fluorescent signal from telomeric PNA probes that hybridize to a given telomere is directly proportional to telomere length, providing a quantitative measurement of telomere length [25]. FISH methods for telomere length measurements can be divided into two groups, namely quantitative (Q)-FISH, based on digital fluorescence microscopy, and Flow-FISH, based on flow cytometry.

### Quantitative Fluorescence *In Situ* Hybridization (Q-FISH) on metaphases

Measurement of telomere length by Q-FISH is based on the quantification of telomeric fluorescent signals derived from PNA probes that hybridize to telomeric repeats, and which are quantified from digital fluorescence microscopy images [25, 33] (Table 1). In a typical telomere Q-FISH image, a double staining can be observed, one for telomeres (e.g., with a telomeric PNA probe labeled with Cy3 and one for chromosomes (e.g.,with 4,6-diamidino-2-phenylindole dihydrochloride [DAPI]) (Figure 5a and d). In fact, chromo-some differential staining represents the key of image analysis procedure, as the chromosome image is used to define the area where the telomere signal is quantified. Individual telomere fluorescent signals are then captured and analyzed, obtaining an estimation of the length of each individual telomere in a given metaphase, which is typically represented by an histogram showing the distribution of telomere length frequencies. Of notice, not all the fluorophores are appropriate for the Q-FISH protocol, as the procedure involves a denaturalization step at 85°C, several washes with 70% formamide, and several dehydration steps with ethanol, and only fluorophores such as Cy3, FITC and TAMRA can be used to label telomeric PNA probes.

An important advantage of the telomere Q-FISH method on metaphases it is high sensitivity, with a low detection limit of only 0.15 kb [25, 34]. This level of sensitivity, allows quantification of the so-called number of “signal-free ends”, namely those chromosome ends with less than <0.15 kb of telomeric repeats and therefore, that could be considered as critically short, a determination which is very important for aging studies as critically short/uncapped telomeres are the ones associated to induction of a DDR, chromosome end-to-end fusions, and the occurrence of aging phenotypes in the context of the telomerase-deficient mouse model [11, 12]. Telomeric Q-FISH on metaphases renders values expressed as arbitrary units of fluorescence (integrated fluorescence intensity values), which can be converted in to Kb by using either plasmids with cloned telomere repeats of defined length [35] or cell lines [36] with known and stable telomere lengths.

In spite of the fact that the Q-FISH on metaphases is the only telomere quantification method capable of measuring all individual telomeres per metaphase (Table 2, Figure 1), is very time consuming and labor-intensive (e.g. it can take a week to process 10 samples) and therefore not suitable for large scale studies (Table 1).

### Quantitative Fluorescence In Situ Hybridization (Q-FISH) on interphasic cells

While the telomere Q-FISH method on metaphases can only be performed on actively proliferating cells as it requires cells at the metaphase stage of the cell cycle, the more recent development of telomere Q-FISH protocols for cells in interphase [37] allow measurement of telomere length in tissue sections or other non-proliferating cell populations, such as postmitotic, differentiated, or senescent cells. A disadvantage of interphase telomere Q-FISH measurements, however, is that it is not possible to measure telomere signals corresponding to individual chromosome ends but instead to aggregations of few telomeres also called “telomere spots” (Table 2 and Figure 1), which precludes the quantification of the so-called “signal free ends”. The quantification of the frequency of low fluorescence telomere spots is, however, a good estimation of the frequency of short telomeres.

### Telomapping

Telomapping is a particular application of the telomere Q-FISH in interphase cells within tissue sections, which is characterized by the generation of color maps that reflect telomere length on a single-cell level within thenormal architecture of the tissue (Figure 5c, f). The original application of telomapping was the finding that tissues show telomere length gradients that reflect on the proliferative and differentiation history of the cells within the tissues [6]. This led to the discovery that known stem cells niches in the mouse are enriched in cells with the longest telomeres within a tissue [6].

More recently, telomapping has been also instrumental for the identification of previously uncharacterized adult stem cell compartments, such as that of the pituitary or human colon [38, 39]. Telomapping is based on high quality confocal images coupled to an accurate highthroughput image analysis platform, which allows quantification of telomere length at the single-cell level in whole-tissue sections providing information on the spatial location of cells within a tissue. Finally, telomapping allows determination of telomere length both per nuclei, as well as at the level of individual telomere spots within each nucleus (Table 2 and Figure 1).

### High-throughput Q-FISH (HT Q-FISH)

The High-throughput (HT) Q-FISH method (HT Q-FISH) is based on the conventional telomere Q-FISH on metaphases but it is performed on interphase nuclei in a 96 well plate format [5] (Table 1 and Figure 4). HT Q-FISH combines, the labeling of telomeres with a PNA probe against the telomeric repeats, and the use of a HT microscopy equipment. Similarly to metaphase Q-FISH, HT Q-FISH is performed with very high stringency hybridization conditions, which only allow detection of perfect TTAGGG repeats, reflecting the length of distal telomeric repeats and not subtelomeric repeats.

HT Q-FISH was validated by comparing the telomere length values obtained with either HT Q-FISH or Q-FISH on metaphases in a panel of human and mouse cell lines [5]. In particular, the mean telomere length values obtained by HT Q-FISH showed an excellent correlation with telomere length values obtained by using conventional QFISH analysis on metaphases, thus indicating a similar accuracy and sensibility. In this manner, HT Q-FISH circumvents the time-consuming steps involving image acquisition and image and data analysis required by the metaphase Q-FISH. Furthermore, HT Q-FISH is amenable to almost any cell type that can be adhered to a support, including lymphoid cells or any other cells isolated from biological fluids. Finally, HT Q-FISH requires low cell numbers going from 10.000 for adherent cells and 70.000 for lymphoid cells (Table 2).

**Figure 3:**
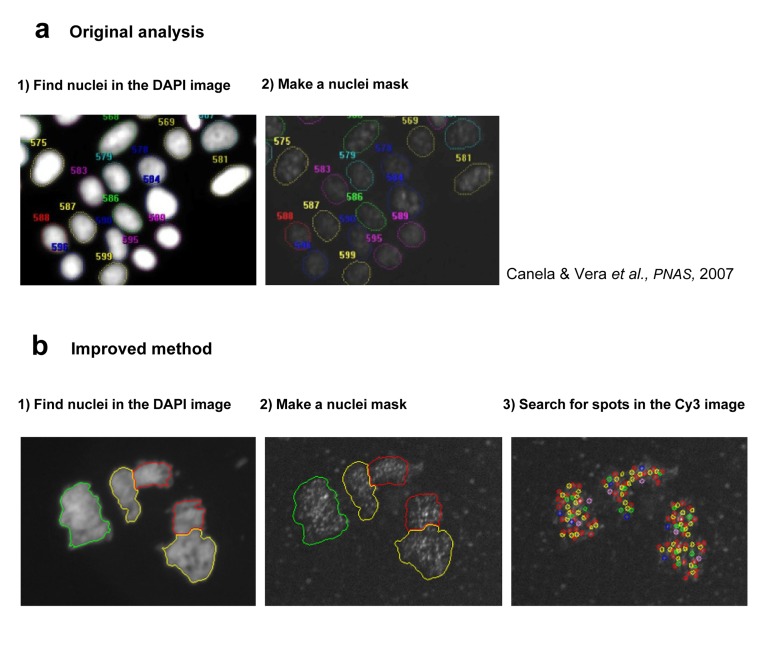
Description of the Image analysis process in original (a) and improved (b) HQ Q-FISH method (**a**) In the original analysis, the DAPI image was used to create a nuclei mask, and all the CY3 inside this mask is quantified obtaining a mean TL per nucleus (**b**) The next step was to search for telomeric spots within the nucleus, and to measure, not the mean TL per nucleus but the TL of each individual spot, solving the main difference with the original QFISH on metaphase spreads.

**Figure 4:**
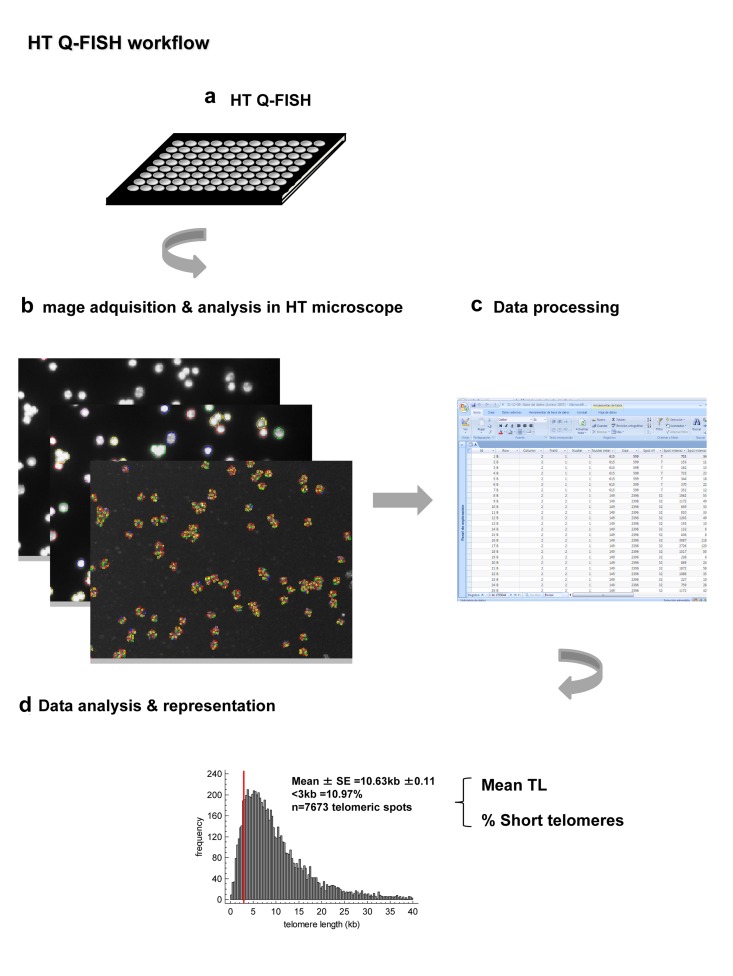
Summary of the main steps of the improved HT Q-FISH method (**a**) HT Q-FISH method is performed on 96 ell plate. Telomeres are labeled with fluorescent PNA probe against the telomeric repeats and nuclei are counterstains with DAPI. Cells are fixed with methanol:acetics. (**b**) Images are acquired and analyzed in a HT confocal microscope. (**c**) The data corresponding to the intensity of each one of the telomere spots are obtained and process. (**d**) A frequency histogram of telomere spots is obtained for each sample, allowing the quantification of the frequency of low intensity telomere spots as estimates of the frequency of short telomeres.

**Figure 5:**
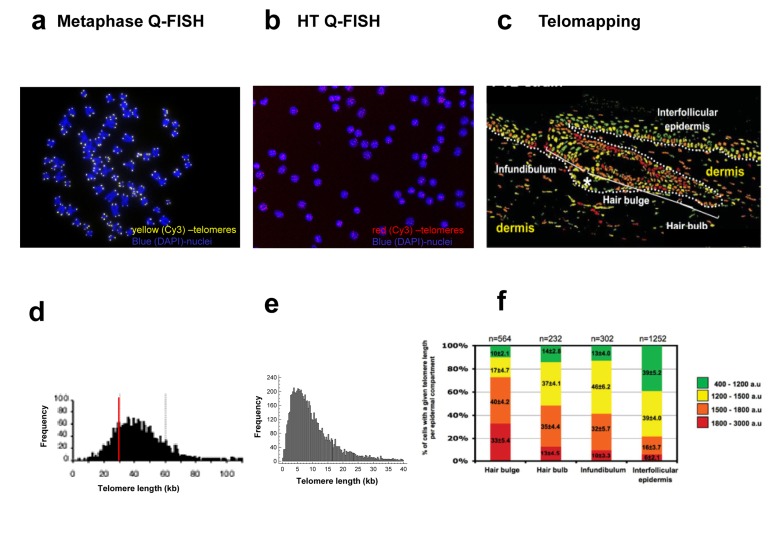
Comparison of the images obtained (a-c) and the output of the data analysis process (d-f) of the different variants of Quantitative FISH methods (**a**) A metaphase image obtained by the regular Q-FISH on metaphases. Telomeres are stained with Cy3 (yellow) and chromosomes are stained with DAPI (blue). Images are acquired with a regular fluorescent microscope (100X augmentation). (**b**) PBMC interphase nuclei image obtained by HT Q-FISH method. Telomeres are stained with Cy3 (red) and chromosomes are stained with DAPI (blue). Images are acquired with an automated HT confocal microscope (40X augmentation). (**c**) Telomere length pseudo-color images obtained by Telomapping of a skin section. Nuclei are colored according to their average telomere fluorescence in arbitrary units (a.u.), from shorter to longer green, yellow, orange and red. Images are acquired with a confocal microscope (20X augmentation). (**d**) Frequency histogram of individual telomeres (kb). 20-30 metaphases per sample are usually analyzed. (**e**) Frequency histogram of individual telomere spots. 100-800 interphases per sample are usually analyzed. (**f)** Telomere length frequency histograms for cells located in the different regions of interest, in this case, in the indicated skin compartments. The number of nuclei analyzed is the corresponding to each region of interest.

The original HT Q-FISH method was able to quantify the mean telomere length of individual nuclei providing frequency histograms of telomere lengths per nuclei, thus allowing the calculation of variables such as the frequency of nuclei with short telomeres in a cell population [5] (Figure 3). A recently improved HT Q-FISH methodology now allows quantification of individual telomere spots within single nucleus (Figure 3, 4 and 5b and e) [21, 40]. Importantly, the HT Q-FISH method is the only highthroughput telomere quantification methodology that allows the calculation of the frequency of short telomeres per cell in large cell populations (Table 2 and Figure 1).

### Flow Fluorescence In Situ Hybridization (Flow-FISH)

The Flow-FISH method consists on labeling of telomeres with a fluorescent PNA probe followed by telomere fluorescence measurements on individual cells by using fluorescence-activated cell sorting (FACS) technology [26] (Table 1). In the Flow-FISH technique, cell suspensions are hybridized with a telomeric fluorescein isothiocyanate (FITC)-conjugated PNA probe and counterstained for DNA to normalize for DNA content. Sample acquisition by flow cytometry provides telomere length values in fluorescence units per counted cell. As with the Q-FISH technique, to obtain absolute telomere length values, external calibration using cell lines with stable and known telomere length is required. Flow-FISH is a powerful method for medium-scale telomere length analysis with potential clinical applications as it is simple and amenable to automation. As an important draw-back, Flow-FISH only provides mean values of telomere length per cell, in contrast to the quantification of individual telomere spot signals in the case of the confocal-microscopy based techniques such as interphase Q-FISH, telomapping or HT Q-FISH (Table 2 and Figure 1).

### Quantitative PCR

Quantitative polymerase chain reaction (qPCR) assay for telomere length determination measures telomere (T) signals and single copy gene (S) signals, in comparison to a reference DNA, to yield relative T/S ratios that are proportional to average telomere length (Table 1). In the original qPCR method, T signals and S signals are quantified in separated wells containing an equivalent sample [28]. Later on, the assay was multiplexed, in the so called monochrome multiplex quantitative PCR method (MMQPCR), in which T and S were quantified in the same well, avoiding significant errors associated to pipetting volume discrepancies between telomere and single copy gene reactions [41]. Like TRF, telomeric qPCR uses genomic DNA to measure telomere length, however, unlike the TRF assay qPCR requires only small amounts of DNA and it is highly amenable to a high-throughput format. An important disadvantage of the qPCR method versus the TRF analysis, relies on the fact that qPCR only renders relative telomere length values and cannot provide absolute telomere length values in kilobases [28]. This problem has been recently circumvented by introducing an oligomere standard to measure absolute telomere length [42]. Finally, and additional shortcoming of the qPCR method is that it can only provide mean telomere length values per cell sample, not being able to determine telomere length per cell, let alone per individual telomere (Table 2 and Figure 1). Thus, qPCR is unable to give an estimation of the percentage of short telomeres in the population, which is an important parameter for assessing telomere functionality.

### Other methods

The increasing interest in telomere length quantification for aging studies has led to the development of new methods to circumvent the above-discussed drawbacks of the most widely used methods, such as the Southern Blot based TRF method. Below, we discuss some of these new methodologies.

The hybridization protection assay (HPA) is a chemiluminescence assay based on the use of an acridinium ester (AE) labeled probe [43]. An alternative method, the hybridization assay is a colorimetric method based on an enzymatic hybridization assay, in which a biotin-coupled-tracer oligonucelotide hybridizes with telomere fragments and the enzymatic reaction is performed with a streptavidin-acetycholinesterase conjugate. Both methods are easy to handle, simple, rapid and quantitative, and eliminate undesired quantification of subtelomeric sequences as it is the case for the TRF analysis. However, as in the case of TRF, they only provide an estimation of the mean telomere length of a cell population, not being able to distinguish individual telomeres or cells.

The primed in situ (PRINS) labeling reaction is based on in-situ hybridization of synthetic oligonucleotides to complementary nucleic acid sequences followed by primer extension in the presence of fluorochrome labeled nucleotides [44]. This technique is complementary to the FISH approaches and allows quantification of individual telomeres, and therefore of the abundance of critically short telomeres. As with Q-FISH, PRINS also requires live cells.

The single telomere analysis, STELA, quantifies individual telomeres and therefore can also estimate the presence of short telomeres [45] (Table 2 and Figure 1). However, because of the need of designing chromosome-specific primers, it can only be applied to few chromosomes [46]. This limitation, has been recently solved with the Universal STELA, which is able to measure the load of short telomeres in a biological sample regardless on which chromosome end the short telomere is located [47]. However, Universal STELA, it is a labor-intensive technique, not amenable to large population studies.

### Different methods provide different information: Choosing the right method

The main objective of telomere length quantification methods is to detect small variations in telomere length between samples, both from different individuals as well as from the same individual over time (longitudinal measurements). The ability to detect small differences in telomere length may be influenced by the measurement technique used, therefore, it is important to select the most sensitive technology for the type of sample available. Depending on the amount of material and the number of samples, detection levels and scalability are also important considerations, respectively.

Regarding the type of sample to be analyzed, Q-FISH, HT Q-FISH, Flow-FISH and PRINS require live cells, such as lymphoid or adherent cells. The rest of the methods, including TRF, STELA and qPCR, do not require live cells as they are performed on DNA.

Regarding the amount of sample material, STELA (0.1 × 106 cells), Q-FISH (~20 metaphases or 30 interphases) or HT Q-FISH (0.01 × 106 cells for adherent cells and 0.07 × 106 cells for lymphoid cells) are ideally suited for low amount of available material. qPCR (20 ng DNA) and Flow-FISH (0.5-2 × 106 cells) require intermediate amounts of material, while and TRF requires the largest amount of cells (106- cells) (Table 2). Regarding the number of samples, as mentioned before, qPCR, HT Q-FISH and Flow-FISH are the best suited for large-scale sample analysis. TRF, Q-FISH on metaphases and STELA are well adapted to small laboratory studies (Table 2 and Figure 1).

An additional important classification of telomere length measurement methods separates them according to the telomere length related variable measured by each method (Table 2 and Figure 1). Some methods, such as TRF or qPCR provide a mean telomere length per sample. STELA quantifies the short telomeres, but it is restricted to several very well characterized chromosome ends and Universal STELA is able to measure the load of short telomeres regardless of chromosome but is not suited to measure the mean telomere length. Other methods provide telomere length distributions based on the mean telomere length of individual cells (Flow-FISH) or based on individual telomeric signal distributions (Q-FISH and Improved HT Q-FISH). These distributions provide large amount of information, most importantly the frequency of nuclei with short telomeres or the frequency of short telomeres. As mentioned before, in terms of biological significance, the ability to quantify the frequency of short telomeres provides the maximum sensitivity. Mean telomere length of individual cell quantification is a good approximation as it allows to calculate the frequency of cells with short telomeres (cells with short mean telomere length); however short telomeres in a cell with long mean telomere length will be undistinguished, and as a consequence, the ability to sense small changes in telomere length is reduced. In all cases, methods that are only able to quantify the mean telomere length of the whole cell population are the less sensible to telomere length changes and will only be able to sense big telomere length differences while ignoring telomere length variability of the different cells in the sample.

### Future perspectives: towards more accurate HT telomere length measurements in human population studies

Although mean telomere length quantification has been widely used in cancer and aging studies, the ability to measure the abundance of short telomeres in high throughput studies has recently emerged. Telomere measurement methods have evolved from cell population analysis (TRF, Figure 7) to individual cell analysis (FISH methods, Figure 7) moving towards more automatized and high-throughput methods. It has been possible to develop high throughput methods that were capable of measuring telomere length of individual cells (Flow FISH and HT Q-FISH, Figure 7), but individual telomere quantification remained for years an exclusive property of metaphase Q-FISH. As discussed here, we have recently overcame this difference by finding the way of quantifying individual telomeric spots within a high throughput platform (Improved HT-QFISH, Figure 7), achieving accuracy and efficiency in terms of time consumption in the same protocol. The ability, of quantifying signal free ends, characteristic of metaphase Q-FISH, is still missing in HT methods, as it is not likely to be practical to work with metaphases in a HT platform. A good compromised have been achieved, however, by HT Q-FISH, increasing by thousands the number of nuclei analyzed, which statistically compensates the technical differences.

**Figure 6:**
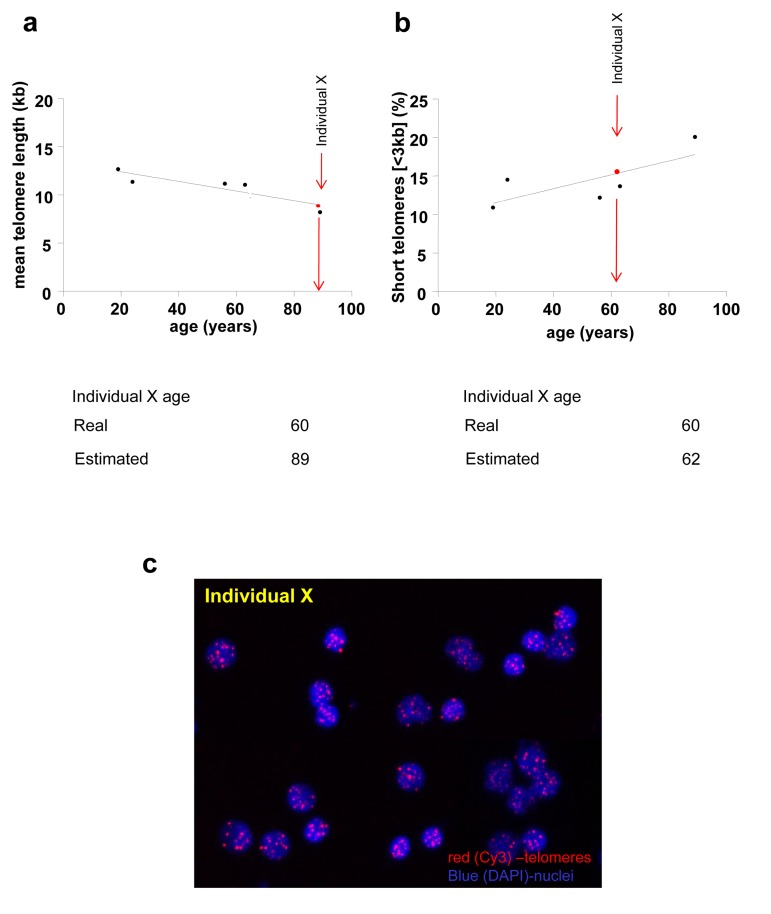
Biological age estimation from a given mean telomere length (a) or a percentage of short telomeres (b) of a given individual (Individual X) Biological age of individual X is estimated extrapolating mean telomere length (**a**) or the percentage of short telomeres (**b)** of individual X in the standard telomere length shorten-ing line based on the telomere length of healthy individuals. Standard line is fitted with 5 individuals for illustrative reasons; real standard lines are not shown. (**c**) Representative HT Q-FISH images of PBMC interphase nuclei. Telomeres are stained with Cy3 (red) and chromosomes are stained with DAPI (blue).

**Figure 7:**
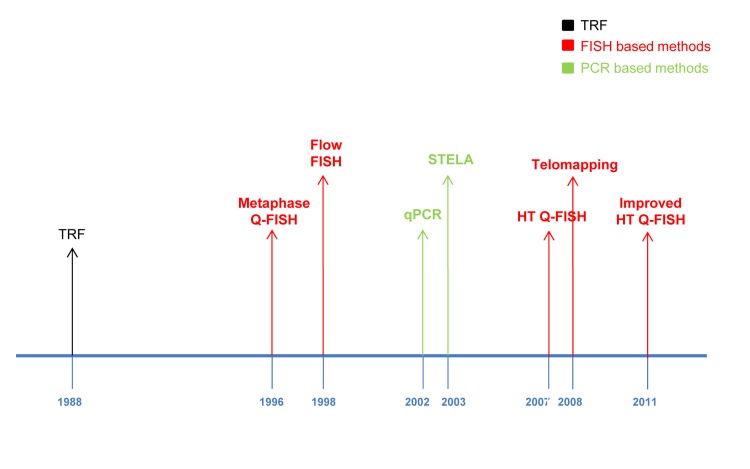
Main telomere length measurement methods timeline: TRF method (black), FISH bases methods (red) and PCR based methods (green).

The ability to measure short telomeres in a high-throughput way opens up exciting possibilities for a shift in human population studies towards more accurate and exhaustive studies. Most of the applications of high throughout telomere length quantification methods are derived from the association between telomere shortening and aging. This association is supported by the fact that the rate of telomere shortening with aging is accelerated in some premature aging syndromes owe to mutations in telomerase such as some cases of Dyskeratosis congenita (DC) or aplastic anemia [48, 49]. Furthermore, shorter telomeres in increasing generation of DC patients have been shown to be associated to disease anticipation [48], indicating a causal association between short telomeres and aging phenotype. In addition, telomere shortening through life is affected by lifestyle associated variables such as smoking, obesity and psychological stress[50-52], suggesting that telomere length may be a good indicator of general health status and the biological age of individuals. Therefore, it is plausible that the variability in telomere length for normal human populations could be determined by various factors, including chronological age, genetics, as well environment and life-habits.

A single telomere length measurement by itself has no biological value, but the comparison with the telomere length of a representative sample of the population. In this regard, the knowledge of telomere length of “healthy” individuals of different ages allows the comparison of a given individual telomere length with his/her age and gender group, providing an idea of his/her general health status or even an estimation of his/her biological age (Figure 6). Moreover, telomere length measured over time (longitudinal studies) may provide a valuable instrument to characterize positive or negative effects of several treatments or changes in lifestyle habits. Additional aging markers could be measured in parallel to telomere length, in the context of future human population studies such as p16 and 53BP1 levels, to be able to define an “aging signature”. Thus, telomere length measurement arises as a new tool for aging and health status characterization with multiple novel applications.
